# The Effect of Adjunctive Mangosteen Pericarp on Cognition in People With Schizophrenia: Secondary Analysis of a Randomized Controlled Trial

**DOI:** 10.3389/fpsyt.2021.626486

**Published:** 2021-06-15

**Authors:** Wolfgang Marx, David R. Skvarc, Mohammadreza Mohebbi, Adam J. Walker, Alcy Meehan, Alyna Turner, Andrea Baker, Seetal Dodd, Sue M. Cotton, James Graham Scott, Bianca E. Kavanagh, Melanie M. Ashton, Ellie Brown, John J. McGrath, Michael Berk, Olivia May Dean

**Affiliations:** ^1^Deakin University, The Institute for Mental and Physical Health and Clinical Translation (IMPACT), School of Medicine, Geelong, VIC, Australia; ^2^Biostatistics Unit, Faculty of Health, Deakin University, Geelong, VIC, Australia; ^3^School of Medicine and Public Health, Faculty of Health and Medicine, The University of Newcastle, Newcastle, NSW, Australia; ^4^Queensland Center for Mental Health Research, The Park Center for Mental Health, Wacol, QLD, Australia; ^5^Center for Youth Mental Health, The University of Melbourne, Parkville, VIC, Australia; ^6^Orygen, Parkville, VIC, Australia; ^7^QIMR Berghofer Medical Research Institute Mental Health Programme, Herston, QLD, Australia; ^8^Metro North Mental Health Service Herston, Herston, QLD, Australia; ^9^Queensland Brain Institute, University of Queensland, St Lucia, QLD, Australia; ^10^National Center for Register-Based Research, Aarhus University, Aarhus, Denmark; ^11^Florey Institute for Neuroscience and Mental Health, University of Melbourne, Parkville, VIC, Australia

**Keywords:** mangosteen, *Mangostana garcinia* Linn., schizophrenia, schizoaffective disorder, psychiatry, mental disorders, cognition

## Abstract

**Background:** Cognitive impairment is prevalent and often highly burdensome in people with schizophrenia. The aim of this study was to investigate if mangosteen (*Garcinia mangostana* Linn.) pericarp extract may be an effective intervention to improve cognitive performance in this population.

**Methods:** This was a secondary analysis of a larger randomized placebo-controlled trial that investigated a 24-weeks intervention of mangosteen pericarp extract supplementation in people diagnosed with schizophrenia. A subset of *n* = 114 participants with completed cognitive outcomes at follow up were included in this analysis. Using the Cogstate Brief Battery, the following cognitive outcomes were assessed: psychomotor function, attention, visual learning and memory (visual and working). Subgroup analyses investigated whether baseline clinical parameters (baseline cognitive functioning, illness severity and duration, depressive symptoms) moderated the relationship between mangosteen pericarp extract intervention and change in cognitive outcomes.

**Results:** There were no significant between-group changes in any cognitive outcomes assessed. Subgroup analysis based on baseline cognition and clinical characteristics did not reveal any significant between-group difference in change.

**Conclusions:** Mangosteen pericarp extract did not affect cognitive outcomes in people with schizophrenia. Further investigation regarding optimal dosing strategies for mangosteen interventions and the testing of additional cognitive domains may be warranted.

**Trial Registration:** ANZCTR.org.au identifier: ACTRN12616000859482, registered 30 June 3 2016.

## Introduction

Cognitive impairment is highly prevalent in people living with schizophrenia, with upwards of 80% experiencing considerable cognitive deficits (1). These wide-ranging cognitive impairments negatively influence daily life, with memory and processing speed most affected (2, 3). Prior reviews have demonstrated the negative effect of this cognitive impairment on functional outcomes including career success and independent living (3). These deficits are not explained by prescribed pharmacotherapy, duration of illness, or psychotic symptoms but rather, are core symptoms of schizophrenia (4). There is limited support for conventional pharmacological interventions improving cognition and functioning in schizophrenia. This is highlighted in a large meta-analysis (*n* = 93 trials) that reported a minor pooled treatment effect (g = 0.10) for global cognition, with no significant effects for any cognitive subdomain and limited support for any one treatment type (5).

Preliminary clinical and preclinical data suggest that mangosteen (*Garcinia mangostana* Linn.) pericarp contains unique bioactive phytochemicals such as flavonoids and xanthones that may beneficially modulate pathways implicated in schizophrenia-related cognitive impairment, including antioxidant, neuroprotective, anti-inflammatory, and mitochondrial-enhancing properties (6). Mangosteen was found to mitigate cognitive deficits and ameliorate oxidative stress within the hippocampus of Flinders Sensitive Line rats (7), and prevented age-related cognitive impairment and increased BDNF levels in older C57BL/6J (B6) mice (8). Similar results were reported in an animal model of Alzheimer's disease wherein mangosteen mitigated scopolamine-induced memory impairment in Morris water maze and passive avoidance tests as well as mitigated oxidative stress (9).

To date, there are no human intervention studies that have investigated the potential effect of mangosteen on cognitive outcomes in people with schizophrenia or otherwise. Hence, due to the need for novel interventions to manage schizophrenia-related cognitive impairment, coupled with the promising pre-clinical efficacy and mechanistic evidence in support of mangosteen, the aims of this study were to:

1. Determine whether 24 weeks of adjunctive mangosteen pericarp extract supplementation affected change in cognitive functioning from baseline (specifically, psychomotor function, attention, visual learning and visual/working memory) compared to placebo,
2. Investigate whether baseline clinical parameters (baseline cognitive functioning, illness severity and duration, depressive symptoms) moderated the relationship between mangosteen pericarp extract intervention and change in cognitive outcomes.

## Methods

This study is a secondary analysis of a randomized controlled trial (ANZCTR.org.au identifier: ACTRN12616000859482) that completed data collection in February 2019. The full study protocol has been published elsewhere (10). Primary analysis of cognitive outcomes were preregistered and the investigation of subgroup responses was conducted as a *post-hoc* subgroup analysis. In brief, this was a 24-weeks double-blind, placebo-controlled (1:1 treatment allocation ratio) randomized clinical trial that was conducted in two sites in Australia (Geelong, Victoria, and Brisbane, Queensland). The timeframe and dose for this study was informed by a previous pilot study (11). Participants provided written informed consent. Human ethics approval was received from Barwon Health Human Research Ethics Committee (HREC), Geelong, Victoria (reference number 15/26); and Metro South Health Service District HREC, Queensland (reference number HREC/16/QPAH/15). Participating institutions included Deakin University, University of Queensland, Barwon Health and the Metro South Health Service.

### Eligibility Criteria

Participants were eligible if they met the following inclusion criteria: aged ≥18 years, diagnosed schizophrenia or schizoaffective disorder using Diagnostic and Statistical Manual of Mental Disorders (fifth edition, DSM-5) diagnostic criteria; scored ≥54 on the PANSS and/or ≥3 on the Clinical Global Impressions severity of illness scale (CGI-S); treatment stable for ≥4 weeks prior to enrolment (if on psychotropic therapy); using effective contraception (if female); able to speak, read, write, and understand the English language; have a current treating physician; and have capacity to consent to the study. Exclusion criteria were the following: known or suspected clinically unstable systemic medical disorder; pregnant or breastfeeding; contraindications or intolerance to mangosteen pericarp or any of the trial preparations; or currently enrolled in another clinical trial.

### Intervention

Participants randomly assigned to the intervention received mangosteen pericarp extract capsules (1,000 mg/days, two 500 mg capsules per day, VitalXan, Adelaide, Australia). Further details of the intervention product are described in the protocol paper (10). Matched placebo tablets were produced to be identical to the intervention in appearance, color and taste. The intervention and placebo were packaged in identical bottles to ensure double-blinding.

### Outcomes

Using the CogState Brief Battery, Maruff et al. (12) delivered on identically configured laptop computers at both sites, the following cognitive outcomes were assessed based on participant performance speed (scored using the mean of the log_10_ transformed reaction times for correct responses) and/or accuracy (scored using the arcsine transformation of the square root of the proportion of correct responses):

*Psychomotor function* was assessed using the speed of performance in the Detection test, which measures processing speed during a simple reaction time design. *Attention* was measured using the speed of performance in the Identification test, which measures attention using a choice reaction time paradigm whereby participants are required to correctly identify the color of flipped cards as quickly as they can. The speed of performance and accuracy scores of the One Card Learning test was used to assess *visual learning and visual memory* using a pattern separation paradigm whereby the participant is asked to identify previously displayed playing cards correctly. *Working memory* was tested using the speed of performance and accuracy scores of the One Back test, which incorporates a n-back paradigm, whereby participants are asked to correctly identify if the current card matches the previously drawn card.

Based on performance on these subtests, two composite outcomes were derived; (i) *Learning-memory* composite, which was derived from the One Back test and One Card Learning test to derive composite accuracy and performance speed outcomes; and (ii) a *Psychomotor composite* score combining performance speed outcomes of the Detection and Identification tests.

To investigate the treatment response based on baseline cognitive function, we defined low baseline cognitive performance as one standard deviation from the mean of the sample due to two considerations. Firstly, a deficit of one standard deviation from the mean performance is characteristic of mild cognitive impairment on the psychomotor composite (13). Second, one standard deviation change is a commonly accepted signifier of cognitive decline over time (14). Treatment response was also assessed according to baseline clinical symptom severity using the Positive and Negative Syndrome Scale (PANSS) total score using cut-offs of >95 (Marked to Severe) (15). Positive (PANSS_P_) and negative (PANSS_N_) sub scores (16), duration of illness (years), and depressive symptoms [Montgomery Åsberg Depression Rating Scale, (MADRS)] were also assessed (17).

### Statistical Methods

Statistical analyses were conducted using IBM® SPSS® Statistics Version 26.0. *P-*values were set at <0.01 to account for multiple comparisons false discovery rate. Participant characteristics were reported as mean (standard deviation) or as a percentage, as appropriate.

Following the data analysis approach of the original RCT, a modified intention to treat was implemented and missing data for participants that completed the Cogstate assessment at follow up were imputed using multiple imputation (five imputations) technique with missing at random assumption. All cognitive outcomes were transformed to standardized z scores for analysis. Generalized estimation equation (GEE) approach with identity link assuming Normal distribution for the outcome was implemented for all main and secondary analyses. The GEE model includes nominal time, nominal group allocation and the two-way interaction between time and group allocation. In this setting, the two-way interaction between time and group allocation estimates the between group differential change from baseline to week 24 in the intervention vs. control group. An unstructured covariance pattern was considered to account for within participants autocorrelation in time. Cohen's d of between group differential change were also calculated. Effect sizes > 0.50 are interpreted as large, effect size of 0.50–0.30 as medium, effect size of 0.30–0.10 as small, and those <0.10 as trivial (18).

## Results

Of the 145 participants recruited to the original study, 114 participants that completed cognitive assessment at baseline and follow up were included in this analysis. The average age of the sample was 39 years (SD = 11.771), and 70% were male (see Table 1). Most participants were Australian born (82%, *n* = 94). Regarding the clinical characteristics of the cohort, most had a diagnosis of schizophrenia (83.5%) with the remainder diagnosed with schizoaffective disorder (16.5%). The average age of formal diagnosis was 25.2 (SD = 8.0) years.

**Table 1:** Participant demographics.

		**Total**	**Placebo**	**Intervention**
**DEMOGRAPHIC**				
Gender %male	*n* (%)	80 (70)	39 (72.2)	41 (67.2)
Age	M (SD)	39 (11.77)	39.06 (12.35)	39.05 (11.36)
Country of Birth (Australian Born)	*n* (%)	94 (82)	44 (81.5)	50 (82)
Aboriginal/Torres Strait Islander	*n* (%)	3 (2.6)	3 (5.6)	0 (0)
**DIAGNOSIS**				
Schizophrenia	*n* (%)	96 (83.5)	44 (81.5)	52 (85.2)
Schizoaffective disorder	*n* (%)	19 (16.5)	10 (18.5)	9 (14.8)
**CLINICAL CHARACTERISTICS**				
Age of diagnosis	M (SD)	25 (8.11)	25.93 (7.62)	24.48 (8.53)
PANSS total	M (SD)	73 (14.004)	69.13 (12.8)	76.52 (14.21)
MADRS	M (SD)	11 (9.02)	9.94 (7.98)	12.3 (9.79)

*MADRS, Montgomery-Asberg Depression Rating Scale; PANSS, Positive and Negative Syndrome Scale*.

### Mangosteen Pericarp Extract Supplementation and Cognitive Outcomes

There was no between-group difference in differential change from baseline to 24 weeks for all cognitive outcomes measured (Table 2). Although the mangosteen intervention reported a greater change for all cognitive outcomes compared to the placebo group, the effect sizes were low for all outcomes (Cohens d < 0.11).

**Table 2:** Mean (±95% confidence intervals) in cognitive outcomes (standardized Z scores) from baseline to end of treatment.

	**Placebo**		**Intervention**			
	**Baseline**	**Follow up**	**Baseline**	**Follow up**	**Between-group change (95% CI)**	**Cohens D**
**DETECTION TEST**						
Speed of performance	−0.13 (−0.52, 0.27)	0.01 (−0.22, 0.24)	−0.009 (−0.37, 0.35)	0.01 (−0.24, 0.27)	−0.12 (−0.65, 0.42)	0.10
**IDENTIFICATION TEST**						
Speed of performance	−0.02 (−0.25, 0.21)	0.0004 (−0.23, 0.23)	0.02 (−0.26, 0.29)	−0.02 (−0.27, 0.23)	−0.05 (−0.38, 0.28)	0.05
**ONE CARD LEARNING TEST**						
Speed of performance	−0.04 (−0.3, 0.22)	−0.0003 (−0.23, 0.23)	0.02 (−0.32, 0.37)	0.003 (−0.25, 0.25)	−0.06 (−0.49, 0.37)	0.05
Accuracy	0.04 (−0.34, 0.43)	0.0003 (−0.22, 0.22)	0.3 (−0.37, 0.97)	0.002 (−0.25, 0.26)	−0.25 (−1.15, 0.64)	0.14
**ONE BACK TEST**						
Speed of performance	0.0001 (−0.23, 0.23)	0.0004 (−0.23, 0.23)	−0.07 (−0.61, 0.47)	−0.02 (−0.3, 0.25)	0.04 (−0.56, 0.64)	0.03
Accuracy	−0.005 (−0.23, 0.22)	0.00004 (−0.23, 0.23)	−0.06 (−0.36, 0.24)	−0.04 (−0.29, 0.21)	0.02 (−0.41, 0.44)	0.02
**LEARNING-MEMORY COMPOSITE**						
Speed of performance	−0.04 (−0.4, 0.32)	0.0002 (−0.37, 0.37)	−0.04 (−0.81, 0.73)	−0.03 (−0.44, 0.37)	−0.03 (−0.83, 0.77)	0.11
Accuracy	0.04 (−0.45, 0.53)	0.0003 (−0.4, 0.4)	0.26 (−0.52, 1.03)	−0.03 (−0.41, 0.34)	−0.25 (−1.32, 0.81)	0.01
**PSYCHOMOTOR COMPOSITE**						
Speed of performance	−0.15 (−0.68, 0.39)	0.01 (−0.39, 0.41)	0.03 (−0.52, 0.58)	0.02 (−0.37, 0.41)	−0.17 (−0.87, 0.54)	0.09

Similarly, subgroup analyses based on low baseline cognition reported no differences in change between groups (Table 3). Further sensitivity analyses based on baseline schizophrenia severity (PANSS total score, negative and positive sub-scores) and duration, as well as depressive symptoms (MADRS), also reported no difference between groups.

**Table 3:** Subgroup analyses of cognitive outcomes (standardized Z scores) from baseline to end of treatment.

	**Baseline cognition**		**Baseline PANSS Total**		**PANSS positive**		**PANSS negative**		**Duration of illness**		**MADRSS**	
	**Normal–High**	**Low**	**Marked to Severe**	**Mild to Moderate**	**High**	**Low**	**High**	**Low**	**>13 years**	**<13 years**	**High**	**Low**
**DETECTION TEST**												
Speed of performance	−0.04 (−0.6, 0.51)	−0.69 (−1.51, 0.13)	0.43 (−4.01, 4.87)	−0.12 (−0.67, 0.42)	0.24 (−0.29, 0.77)	−0.55 (−1.6, 0.49)	0.11 (−0.46, 0.68)	−0.38 (−1.31, 0.56)	−0.45 (−1.36, 0.46)	0.22 (−0.4, 0.84)	−0.41 (−1.3, 0.48)	0.2 (−0.42, 0.81)
**IDENTIFICATION TEST**												
Speed of performance	−0.08 (−0.39, 0.24)	0.59 (−0.45, 1.63)	−1.19 (−4.23, 1.85)	0.004 (−0.28, 0.29)	0.03 (−0.46, 0.52)	−0.14 (−0.59, 0.31)	−0.03 (−0.53, 0.48)	−0.09 (−0.49, 0.31)	−0.09 (−0.57, 0.39)	−0.02 (−0.43, 0.4)	−0.11 (−0.57, 0.35)	−0.02 (−0.46, 0.42)
**ONE CARD LEARNING TEST**												
Speed of performance	−0.19 (−0.6, 0.23)	0.52 (−0.74, 1.77)	−1 (−4.2, 2.2)	−0.0005 (−0.41, 0.41)	0.07 (−0.55, 0.7)	−0.28 (−0.8, 0.24)	−0.04 (−0.6, 0.52)	−0.08 (−0.71, 0.55)	−0.31 (−0.86, 0.24)	0.23 (−0.39, 0.86)	−0.13 (−0.8, 0.53)	−0.01 (−0.56, 0.54)
Accuracy	−0.57 (−3.35, 2.2)	−0.09 (−0.95, 0.77)	−3.91 (−10.43, 2.61)	−0.07 (−0.92, 0.78)	−0.38 (−2.03, 1.28)	−0.1 (−1.15, 0.95)	−0.82 (−1.99, 0.34)	0.45 (−1.31, 2.22)	−0.15 (−1.06, 0.77)	−0.35 (−1.76, 1.05)	−0.44 (−2.53, 1.65)	−0.06 (−1.46, 1.33)
**ONE BACK TEST**												
Speed of performance	−0.02 (−0.99, 0.95)	0.13 (−0.88, 1.13)	−0.44 (−3.01, 2.13)	0.09 (−0.5, 0.67)	−0.03 (−0.95, 0.88)	0.08 (−0.53, 0.69)	−0.1 (−1.11, 0.91)	0.22 (−0.42, 0.85)	0.2 (−0.68, 1.09)	−0.16 (−0.99, 0.68)	−0.26 (−0.91, 0.39)	0.36 (−0.57, 1.29)
Accuracy	−0.45 (−1.77, 0.88)	0.06 (−0.34, 0.46)	−0.65 (−2.39, 1.09)	0.05 (−0.38, 0.49)	0.04 (−0.59, 0.67)	0.03 (−0.55, 0.6)	0.07 (−0.57, 0.71)	−0.05 (−0.55, 0.46)	0.55 (−0.07, 1.17)	−0.49 (−1.13, 0.15)	−0.13 (−0.6, 0.35)	0.18 (−0.51, 0.88)
**LEARNING–MEMORY COMPOSITE**												
Speed of performance	−0.02 (−0.99, 0.95)	0.13 (−0.88, 1.13)	−1.41 (−6.54, 3.72)	0.06 (−0.67, 0.79)	0.04 (−1.05, 1.14)	−0.18 (−1.14, 0.77)	−0.14 (−1.46, 1.18)	0.08 (−0.83, 0.98)	−0.13 (−1.2, 0.95)	0.09 (−1.02, 1.2)	−0.39 (−1.35, 0.56)	0.34 (−0.86, 1.55)
Accuracy	−1.41 (−3.78, 0.95)	0.31 (−0.7, 1.31)	−4.74 (−11.9, 2.41)	−0.03 (−1.06, 1.01)	−0.4 (−2.27, 1.48)	−0.07 (−1.41, 1.26)	−0.79 (−2.17, 0.6)	0.12 (−1.44, 1.67)	0.39 (−0.83, 1.61)	−0.95 (−2.56, 0.67)	−0.6 (−2.85, 1.65)	0.12 (−1.44, 1.67)
**PSYCHOMOTOR COMPOSITE**												
Speed of performance	−0.23 (−0.99, 0.53)	0.1 (−1.24, 1.44)	−0.63 (−7.16, 5.9)	−0.13 (−0.82, 0.55)	0.3 (−0.5, 1.1)	−0.65 (−1.89, 0.58)	0.12 (−0.7, 0.94)	−0.51 (−1.65, 0.64)	−0.53 (−1.67, 0.6)	0.19 (−0.65, 1.04)	−0.53 (−1.62, 0.56)	0.17 (−0.72, 1.07)

*Data presented as between-group change (95% CI)*.

## Discussion

This is the first study to investigate the effect of mangosteen pericarp extract on cognition in people with schizophrenia. Despite promising preclinical and mechanistic data (6), there was no significant difference in treatment effect compared to placebo, and the results do not support this intervention as an effective therapy for cognitive outcomes in people with schizophrenia. Our findings, together with the finding that mangosteen pericarp extract did not significantly affect between-group differences in change in schizophrenia symptom scores (as measured by the PANSS) (19), weaken the hypothesis that mangosteen pericarp extract has clinical utility for those with schizophrenia.

The results of this trial are also in line with previous nutraceutical interventions for schizophrenia-related cognitive performance (20). A recent review reported that omega-3 fatty acids and taurine failed to improve any measure of cognitive performance (20). N-acetyl cysteine improved some individual cognitive domains, but not global cognition (20). Furthermore, a previous trial that investigated another polyphenol intervention, resveratrol, in people with schizophrenia also reported no significant improvement (21).

The results of this study are in contrast to the extant polyphenol literature in other populations, which has reported improved cognitive outcomes in other populations, including in healthy adults and people with mild cognitive impairment (22, 23). While mangosteen pericarp is rich in polyphenol compounds, the biological properties of the diverse range of polyphenol compounds are not uniform, and so other polyphenol interventions that have demonstrated improvements in other conditions may act on different biological pathways to those polyphenols included in mangosteen pericarp.

While we explored potential baseline factors to identify possible sub-populations that may display greater treatment response, an additional potential explanation for the null findings is that unexplored factors may influence treatment response. In particular, inter-individual differences in the metabolism and pharmacokinetics has been identified for other polyphenol compounds (24). For example, the metabolism of ellagitannins, found in high concentrations in pomegranate husk and juices, is greatly influenced by individual gut microbiota composition (24). The limited studies that have investigated the bioavailability and pharmacokinetics of mangosteen polyphenols also indicate high inter-individual variability with marked variation in the area under the curve (762–4,030 nmol/L × h) of the primary polyphenolic compound, α-mangostin, in serum (25). A related consideration is the need for further investigation of optimal dosing regimens. A previous study reported that roughly 2% of consumed mangosteen polyphenols were absorbed (25), suggesting low bioavailability. Similar low absorption rates have been reported for other polyphenol compounds such as resveratrol and curcumin where novel methods to improve bioavailability have been introduced (26, 27).

These factors speak to the difficulty and complexity of nutraceutical research where the bioavailability and treatment response is potentially modulated by several factors unique to nutraceutical interventions. These factors include the absorption and treatment response of some dietary compounds, including polyphenol compounds, which can be modulated by the food matrix and degree of processing of the nutraceutical formulation. For example, polyphenols derived from apples had a substantially different effect on gene expression depending on the degree of processing (whole apple vs. puree vs. extract) (28). Furthermore, interindividual differences in pathways such as inflammation have also been shown to modulate treatment response to nutraceuticals in psychiatry (29), suggesting that subpopulations may be more amenable to some nutraceutical interventions than others. Related to this is the role of nutrient deficiency and sufficiency in modulating treatment response, whereby nutraceuticals such as vitamin D appear to have a differential treatment effect on depression depending on the baseline serum levels of vitamin D (30). Similarly, due to the presence of some nutraceutical compounds in commonly consumed food items, it is conceivable that some participants may already be consuming higher quantities of nutraceuticals including polyphenols through their habitual diet and that this may in turn, affect individual treatment response. Novel study design features including the measurement of baseline biomarkers, habitual diet, and consideration for the food matrix may improve treatment efficacy in future trials.

Strengths of this study include the rigorous study design, which incorporated double-blinding and placebo control features. The cognitive outcomes of this study were also assessed using a widely-used and validated cognitive battery. Furthermore, adherence, as assessed by pill counts at follow up, was high (94% adherence rate). We acknowledge the following limitations to this analysis. First, while this study was statistically powered based on the primary outcome of the original study (PANSS Total), the subgroup analyses conducted as part of this analysis are likely underpowered. More extensive studies may provide sufficient sample sizes to detect small treatment differences that were not able to be detected in the current analysis. Second, while we included a widely-used and validated tool to measure cognitive outcomes (CogState Brief Battery), this task configuration measures a limited sample of cognitive domains, and so additional cognitive domains may be worthy of future investigation. For example, reasoning/problem solving and social cognition, as recommended by the MATRICS cognitive battery initiative, or the expanded schizophrenia-specific battery developed by CogState (31).

## Conclusion

Despite promising pre-clinical evidence suggesting a therapeutic effect, this study reports that mangosteen pericarp supplementation did not improve cognitive outcomes in people with schizophrenia. While baseline clinical and cognitive factors did not alter this result, potential inter-individual differences in metabolism require further exploration. Furthermore, further pre-clinical investigation of mangosteen pericarp supplementation may be warranted to identify pharmacologically active compounds and ensure that they are present in the formulation and bioavailable with oral dosage.

## Data Availability Statement

The raw data supporting the conclusions of this article will be made available by reasonable request to authors.

## Ethics Statement

The studies involving human participants were reviewed and approved by Human ethics approval was received from Barwon Health Human Research Ethics Committee (HREC), Geelong, Victoria, (reference number 15/26); and Metro South Health Service District HREC, Queensland (reference number HREC/16/QPAH/15). The patients/participants provided their written informed consent to participate in this study.

## Author Contributions

WM led all phases of this study. DS, AW, and OD informed the analysis. OD supervised all phases of the study. MM supervised the statistical analysis. AT, AB, OD, AW, SD, SC, JS, BK, MA, EB, JM, and MB were all involved in design, data collection, and/or analysis of the original clinical trial. All authors provided input to the development of the manuscript.

## Conflict of Interest

AT has received grant/research support from the National Health and Medical Research Council (NHMRC), AMP Foundation and Deakin University. OD was a R.D. Wright NHMRC Biomedical Career Development Fellow (APP1145634) and has received grant support from the Brain and Behavior Foundation, Simons Autism Foundation, Stanley Medical Research Institute, Deakin University, Lilly, NHMRC and ASBDD/Servier. She has also received in kind support from BioMedica Nutraceuticals, NutritionCare and Bioceuticals. AW has previously received grant/research support from a Trisno Family Fellowship and Deakin University. SD has received grant support from Stanley Medical Research Institute, NHMRC, Beyond Blue, ARHRF, Simons Foundation, Geelong Medical Research Foundation, Fondation FondaMental, Eli Lilly, Glaxo SmithKline, Organon, Mayne Pharma and Servier. He has received speaker's fees from Eli Lilly, advisory board fees from Eli Lilly and Novartis and conference travel support from Servier. SC was supported by a NHMRC Senior Research Fellowship (APP1136344). SC has received grant support from the NHMRC, the Stanley Medical Research Institute, BeyondBlue, Movember, The University of Melbourne, Australian Catholic University, ARHRF, and Mental Illness Research Fund (Victoria Department of Human Services). JS was supported by a National Health and Medical Research Council Practitioner Fellowship Grant (APP1105807), has been a speaker for Janssen Cilag, Lundbeck, Servier, and Shire Pharmaceuticals, and served as a consultant to Janssen Cilag, Lundbeck and Roche. BK has received grant/research support from the Australian Postgraduate Research Training Scholarship, Deakin University, Ian Scott Mental Health Ph.D. Scholarship, Australian Rotary Health, and the International Society for the Study of Personality Disorders. MA has received grant/research support from Deakin University, Australasian Society for Bipolar Depressive Disorders, Lundbeck, Australian Rotary Health, Ian Parker Bipolar Research Fund, Cooperative Research Center for Mental Health and PDG Geoff and Betty Betts Award from Rotary Club of Geelong. EB has received grant/research support from the University of Melbourne, the Australian Department of Health, Western Victoria PHN, and the University of the West of England, UK. MB was supported by a NHMRC Senior Principal Research Fellowship (1059660 and 1156072). MB has received Grant/Research Support from the NIH, Cooperative Research Centre, Simons Autism Foundation, Cancer Council of Victoria, Stanley Medical Research Foundation, Medical Benefits Fund, National Health and Medical Research Council, Medical Research Futures Fund, Beyond Blue, Rotary Health, A2 milk company, Meat and Livestock Board, Woolworths, Avant and the Harry Windsor Foundation, has been a speaker for Abbot, Astra Zeneca, Janssen and Janssen, Lundbeck and Merck and served as a consultant to Allergan, Astra Zeneca, Bioadvantex, Bionomics, Collaborative Medicinal Development, Janssen and Janssen, Lundbeck Merck, Pfizer and Servier—all unrelated to this work. WM was currently funded by an Alfred Deakin Postdoctoral Research Fellowship and a Multiple Sclerosis Research Australia early-career fellowship. WM has previously received funding from the Cancer Council Queensland and university grants/fellowships from La Trobe University, Deakin University, University of Queensland, and Bond University, received industry funding and has attended events funded by Cobram Estate Pty. Ltd, received travel funding from Nutrition Society of Australia, received consultancy funding from Nutrition Research Australia, and has received speakers honoraria from The Cancer Council Queensland and the Princess Alexandra Research Foundation. MM has received Grant/research support from NHMRC, Deakin University School of Medicine, Deakin Biostatistics Unit, Institute for Mental and Physical Health and Clinical Translation, and Medibank Health Research Fund. The remaining authors declare that the research was conducted in the absence of any commercial or financial relationships that could be construed as a potential conflict of interest. The reviewer NN declared a past co-authorship with four of the authors WM, OD, MA, and MB to the handling editor.
